# Supplementary material for: Somatic mutation profiling, tumor-infiltrating leukocytes, tertiary lymphoid structures and PD-L1 protein expression in HER2-amplified colorectal cancer
Source: PeerJ. 2023 May 2;11:e15261. doi: 10.7717/peerj.15261 (PMC10162038; doi:10.7717/peerj.15261)
Supplement: Supplemental Information 2 [file peerj-11-15261-s002.doc]

1. targeted therapy, prognosis, and resistance-related genes

ABL1、ABRAXAS1、AKT1、AKT2、AKT3、ALK、APC、AR、ARAF、ARID1A、ATM、ATR、AURKA、BARD1、BCL2L11、BRAF、BRCA1、BRCA2、BRIP1、BTK、CCND1、CCND3、CD274、CDK12、CDK4、CDK6、CDKN1B、CDKN2A、CDKN2B、CHD1、CHEK1、CHEK2、CRBN、CSF1R、CTNNB1、DDR2、DNMT3A、EGFR、EPCAM、EPHA2、EPHA3、ERBB2、ERBB3、ERBB4、ERCC3、ERRFI1、ESR1、EZH2、FANCE、FANCL、FAT1、FBXW7、FGF3、FGF4、FGFR1、FGFR2、FGFR3、FGFR4、FLCN、FLT1、FLT3、FLT4、FOXA1、FRS2、GEN1、GLI1、GLI2、GLI3、GNA11、GNAQ、GNAS、HDAC2、HGF、HOXB13、HRAS、IDH1、IDH2、IGF1R、IGF2、IL7R、INPP4B、JAK1、JAK2、JAK3、KDR、KEAP1、KIT、KRAS、LRP1B、MAP2K1、MAP2K2、MCL1、MDM2、MDM4、MET、MLH1、MLH3、MRE11、MSH2、MSH6、MTOR、MYC、MYCN、NBN、NF1、NF2、NFKBIA、NKX2-1、NRAS、NRG1、NTRK1、NTRK2、NTRK3、PALB2、PBRM1、PCDH9、PDCD1LG2、PDGFRA、PDGFRB、PIK3CA、PIK3R1、PIK3R2、PLCG2、PLXNA1、PML、PMS2、POLD1、POLE、PPP2R2A、PTCH1、PTEN、RAC1、RAD50、RAD51、RAD51B、RAD51D、RAD54L、RAF1、RARA、RB1、RET、RICTOR、RNF43、ROS1、RPTOR、RXRA、SETD2、SMARCA4、SMARCB1、SMO、SRC、STAG2、STK11、SYK、TERT、TP53、TSC1、TSC2、VEGFA、VHL、ZBTB16、ZNRF3

1. DNA-damage repair related genes

ABRAXAS1、AEN、ALKBH2、ALKBH3、APEX1、APEX2、APLF、APTX、ATM、ATR、ATRIP、ATRX、BARD1、BLM、BRCA1、BRCA2、BRIP1、CCNH、CDK12、CDK7、CENPS、CETN2、CHAF1A、CHEK1、CHEK2、CLK2、CUL3、CUL4A、CUL5、DCLRE1A、DCLRE1B、DCLRE1C、DDB1、DDB2、DMC1、DNTT、DUT、EME1、EME2、ENDOV、ERCC1、ERCC2、ERCC3、ERCC4、ERCC5、ERCC6、ERCC8、EXO1、FAAP100、FAAP20、FAAP24、FAN1、FANCA、FANCB、FANCC、FANCD2、FANCE、FANCF、FANCG、FANCI、FANCL、FANCM、FEN1、GEN1、GTF2H1、GTF2H3、GTF2H4、GTF2H5、H2AFX、HELQ、HES1、HFM1、HLTF、HMGB1、HUS1、IDH1、LIG1、LIG3、LIG4、MAD2L2、MBD4、MDC1、MGMT、MLH1、MLH3、MMS19、MNAT1、MPG、MPLKIP、MRE11、MSH2、MSH3、MSH4、MSH5、MSH6、MUS81、MUTYH、NABP2、NBN、NEIL1、NEIL2、NEIL3、NHEJ1、NTHL1、NUDT1、OGG1、PALB2、PARP1、PARP2、PARP3、PARP4、PCNA、PER1、PMS1、PMS2、PNKP、POLB、POLD1、POLD3、POLD4、POLE、POLE2、POLE3、POLE4、POLG、POLH、POLI、POLK、POLL、POLM、POLN、POLQ、PPP4R1、PPP4R2、PPP4R4、PRKDC、PRPF19、PTEN、RAD1、RAD18、RAD23A、RAD23B、RAD50、RAD51、RAD51B、RAD51C、RAD51D、RAD52、RAD54B、RAD54L、RAD9A、RAD9B、RBBP8、RBX1、RDM1、RECQL、RECQL4、RECQL5、REV1、REV3L、RFC1、RFC2、RFC3、RFC4、RFC5、RIF1、RMI1、RMI2、RNF168、RNF4、RNF8、RPA1、RPA2、RPA3、RPA4、RRM2B、SEM1、SETMAR、SHPRH、SLX1A、SLX4、SMARCA4、SMUG1、SPO11、SPRTN、TDG、TDP1、TDP2、TELO2、TOP3A、TOP3B、TOPBP1、TP53、TP53BP1、TREX1、TREX2、UBE2A、UBE2B、UBE2N、UBE2T、UBE2V2、UNG、USP1、UVSSA、WDR48、WRN、XAB2、XPA、XPC、XRCC1、XRCC2、XRCC3、XRCC4、XRCC5、XRCC6

1. human leukocyte antigen genes

HLA-A、HLA-B、HLA-C

1. chemotherapy-related genes

CYP2C19、DPYD、TPMT、UGT1A1

1. genetic susceptibility-related genes

AKT1、ALK、APC、ARAF、ARID1A、ATM、ATR、AXIN2、BAP1、BARD1、BLM、BMPR1A、BRAF、BRCA1、BRCA2、BRIP1、CCND1、CD274、CDC73、CDH1、CDK4、CDK6、CDKN2A、CHEK1、CHEK2、CTNNB1、DDR2、DICER1、EGFR、EPCAM、ERBB2、FANCA、FANCC、FANCD2、FANCG、FBXW7、FGF19、FGFR1、FGFR2、FGFR3、FH、FLCN、FLT3、HRAS、JAK2、KDR、KIT、KRAS、LRP1B、MAP2K1、MAP2K2、MEN1、MET、MITF、MLH1、MRE11、MSH2、MSH6、MTOR、MUTYH、NBN、NF1、NF2、NRAS、NTRK1、NTRK2、PALB2、PDCD1LG2、PDGFRA、PIK3CA、PMS2、POLD1、POLE、PRKAR1A、PRSS1、PTCH1、PTEN、RAD50、RAD51C、RAD51D、RAF1、RB1、RET、ROS1、SDHA、SDHB、SDHC、SDHD、SMAD4、SMARCA4、SMARCB1、SMO、SPTA1、STK11、SUFU、TP53、TSC1、TSC2、VEGFA、VHL、WT1、XRCC2。
